# EFFECTS OF ACUTE AND CHRONIC CANNABIS/CANNABINOIDS ON COGNITIVE FUNCTION ACROSS THE LIFESPAN

**DOI:** 10.1093/geroni/igad104.0034

**Published:** 2023-12-21

**Authors:** Barry Setlow, Sabrina Zequeira, Emely Gazarov, Jennifer Bizon

**Affiliations:** University of Florida, Gainesville, Florida, United States; University of Florida, Gainesville, Florida, United States; University of Florida, Gainesville, Florida, United States; University of Florida, Gainesville, Florida, United States

## Abstract

Cannabis and cannabinoids such as THC (the major psychoactive component of cannabis) tend to impair cognitive performance, but almost all research on this topic has been conducted in young adults. Given that many aged individuals already exhibit cognitive deficits, it is important to determine how cannabis/cannabinoids affect cognition in this population. We used an animal model (young adult and aged rats) to evaluate the effects of both acute cannabis smoke and chronic oral THC on several tests of cognitive function. Both acute smoke (males only) and chronic THC (both sexes) enhanced working memory in aged rats, but neither affected young adults. In contrast, both regimens tended to impair performance on tests of spatial/episodic memory in an age-independent manner. Results suggest that although cannabis has potential for cognitive benefits in aging, its effects likely depend on both the cognitive domain tested and the manner in which it is administered.

